# Cats on dry kibble diet have significantly different microbiome than those on canned wet food

**DOI:** 10.1038/s44433-025-00001-6

**Published:** 2026-03-26

**Authors:** Celeste Allaband, Holly H. Ganz, Connie A. Rojas, Rob Knight

**Affiliations:** 1 Department of Pediatrics, University of California San Diego, La Jolla, CA USA; 2 AnimalBiome, Oakland, CA USA; 3 Center for Microbiome Innovation, University of California San Diego, La Jolla, CA USA; 4 Department of Computer Science and Engineering, University of California San Diego, La Jolla, CA USA; 5 Shu Chien-Gene Lay Department of Bioengineering, University of California San Diego, La Jolla, CA USA; 6 Halıcıoğlu Data Science Institute, University of California San Diego, La Jolla, CA USA

**Keywords:** Microbiome, Clinical microbiology

## Abstract

Domestic cats (*Felis catus*) are understudied regarding how commercial diets impact their gut microbiomes. Here, we reanalyzed the 16S rRNA gene (V4) amplicon sequencing Kittybiome dataset, using new tools and techniques. Results demonstrated significant microbial composition differences between cats eating commercial dry kibble diets and those eating canned wet food. Kibble-fed cats showed enriched *Prevotella*, *Bifidobacterium*, and *Megamonas* amplicon sequencing variants (ASVs), linked to carbohydrate metabolism and metabolic disease.

## Introduction

The diverse community of bacteria in the gut of mammals plays a crucial role in maintaining overall health^1,2^. Just like many other mammalian species, feline gut microbiomes are strongly influenced by diet, age, environmental exposures, and many pharmaceuticals^3–5^. Numerous studies have shown the strong effect of dietary macronutrient contents on community composition of the distal gut microbiota in every species studied. Domestic cats are obligate carnivores with a unique need for additional taurine in their diet, which is reflected in the formulation of commercial cat food diets^6,7^. Here, we focus on the two most common types of commercial cat diet formulations, dry kibble and canned wet food. Dry kibble diets are typically higher in carbohydrates because they are used to provide structural support during the extruding process^8^. Carbohydrates encompass simple sugars, starches, as well as fiber. The average carbohydrate content of dry grocery-brand cat foods is approximately 45%, whereas moist grocery-brand cat foods is approximately 10%^9^. Thus, cats on the average dry kibble food are generally on a higher carbohydrate diet than cats on canned wet food. However, the extent to which dietary formulation impacts cat gut microbiome composition is relatively unknown, and if it is linked to any feline health parameters, such as metabolic disease.

Following a similar model as the NIH-funded Human Microbiome Project (HMP) and the citizen-science-led American Gut Project (AGP), AnimalBiome has been accumulating samples and 16S rRNA gene (V4) amplicon sequencing data from pet felines since their company was founded in 2016. We re-analyzed a stringently selected subset of 172 total owner-reported healthy individuals that was previously published^10^ using a newer bacterial reference database (Greengenes2^11^) and advanced methods with a focus on the two most common commercial cat food formulations, dry kibble and canned wet food. We provide a detailed analysis of the bacterial taxa enriched in kibble-fed cats and discuss the potential implications for feline health.

The previous analysis^10^ focused on identifying the predominant bacterial groups present in the fecal microbiomes of healthy pet domestic cats, with brief mention of additional factors. For this analysis, we exclusively focused on diet and conducted a more detailed analysis of the impact of the two most common diet formulations for cats—dry kibble and canned wet food—on the fecal microbiome. In addition, the previous analysis used a non-phylogenetic beta diversity metric, Bray–Curtis, to evaluate differences in microbial composition. Because the addition of phylogenetic information improves the effect size and classification accuracy of beta diversity metrics^12^, we used the new phylogenetic metric, phyloRPCA. This metric uses both phylogeny and robust centered log-ratio (rclr) transformed abundances to assess microbial composition differences. The rclr transformation better accounts for the sparsity and compositionality of microbiome data^12,13^. Furthermore, we used a relatively new reference database, Greengenes2^11^, that includes updated phylogenetic tree structure and taxonomic nomenclature. Using these methods allowed us to find a clearer underlying pattern in the data based on diet formulation.

**Fig. 1:**
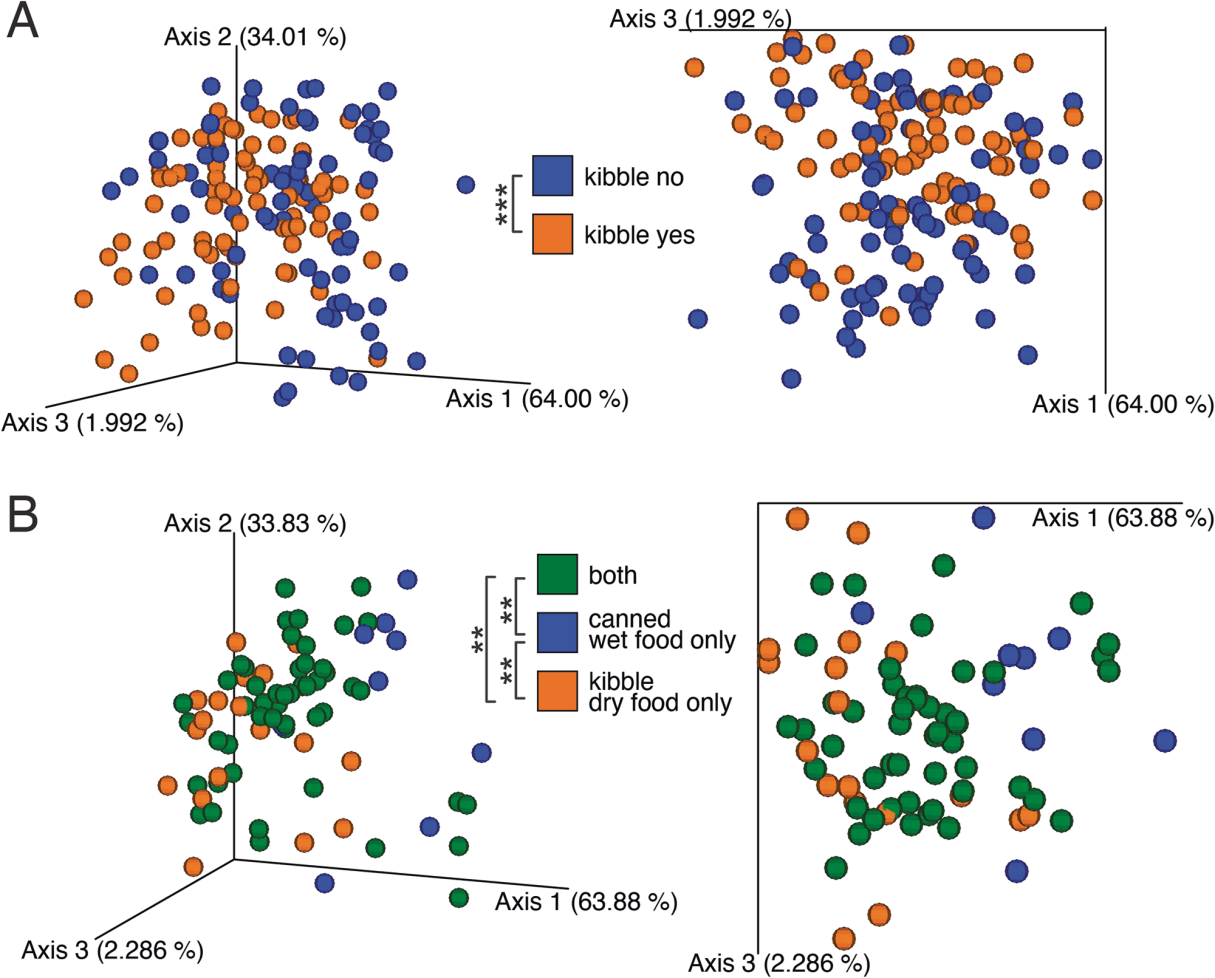
Re-analysis of KittyBiome dataset using phylogenetic robust rclr-based methods (phyloRPCA). **A** PhyloRPCA beta diversity PCoA plot colored by whether the cat owner reported whether they fed the cat any dry kibble food or not. Samples with other responses or unknowns were excluded (*N* = 156). **B** PhyloRPCA beta diversity PCoA plot colored by if the cat owner reported they fed the cat dry kibble only, canned wet food only, or both. Samples with other responses or unknowns excluded (*N* = 77). Left and right of each panel represent two views of the same 3D plot. Significance determined by pairwise PERMANOVA. **p < 0.01, ***p < 0.001.

Mirroring the original findings, cats consuming any dry kibble exhibited significantly lower Shannon alpha diversity compared to those exclusively on wet food, which displayed the highest diversity among all dietary groups (Fig. S1). PhyloRPCA analysis revealed that cats eating at least some dry kibble had significantly different fecal microbial populations compared to cats that reportedly ate no dry food (PERMANOVA, pseudo-*F* 12.9, *p* < 0.001) (Fig. 1A). In addition, when we account for samples from felines that were fed either a dry kibble diet alone, a canned wet diet alone, or both—excluding samples where it could not be determined—there is significant separation of the feline microbiome by diet formulation type (PERMANOVA, pseudo-*F* 5.2, *p* < 0.001) (Fig. 1B). This was especially evident between cats fed dry kibble only compared to cats fed a canned wet food diet only (PERMANOVA, pseudo-*F* 11.6, *p* = 0.004). This indicates that diet formulation type is a significant factor affecting the microbiome composition of domestic cats.

**Fig. 2:**
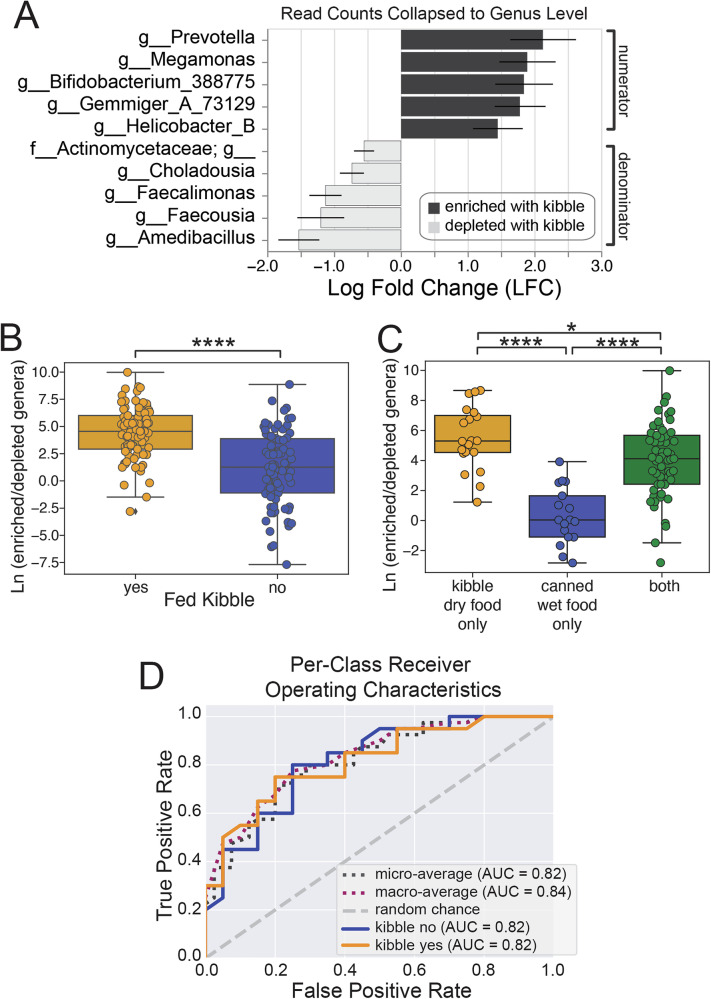
KittyBiome dataset reanalysis differential abundance, log ratio, and random forest analysis. **A** After collapsing to the genus level, ANCOM-BC differential abundance analysis of samples from cats that were or were not fed a dry kibble diet. Results shown had log fold change >0.5 and *p* value < 0.05. Next, a log ratio was created with the numerator comprised of the ASVs from the genera found to be enriched when fed a kibble diet by ANCOM-BC and the denominator those genera found to be depleted, and calculated on a per-sample basis. Using boxplot/swarmplots, the resulting log ratio was examined by **B** if the cat was fed any kibble diet (*N* = 156) and **C** what type of diet(s) the cat was known to consume (*N* = 77). Statistical significance was determined by a two-sided Mann–Whitney *U* test with Benjamini–Hochberg FDR correction. **D** Random forest analysis receiver operating characteristic (ROC) curves. AUC area under the curve. *p < 0.05, ****p < 0.0001.

ANCOM-BC was used to identify differentially abundant genera between cats fed a dry kibble diet or not (log fold change >0.5, *p* value < 0.05) (Fig. 2A). A natural log ratio of all the amplicon sequencing variants (ASVs) belonging to those differentially abundant genera, with the enriched genera in the numerator and depleted genera in the denominator, confirmed a significant difference between those cats fed a dry kibble diet compared to those who did not (two-sided Mann–Whitney *U*, *p* = 2.33 × 10^−8^) (Fig. 2B). This log ratio also was able to significantly separate cats that were fed dry kibble only, canned wet food only, and fed both diets from each other (two-sided Mann–Whitney *U* with Benjamini–Hochberg FDR correction, kibble only vs. wet food only *p* = 2.46 × 10^−6^, kibble only vs. both types *p* = 0.025, wet food only vs. both types *p* = 2.46 × 10^−6^) (Fig. 2C). Cats fed only dry kibble had a median log ratio value about 5 log folds higher than those receiving canned wet food only, indicating increased amounts of reads from those ASVs in the numerator relative to those in the denominator.

Random Forest analysis (Fig. 2D) also showed a strong predictive ability (AUC = 0.82) in separating samples from cats fed any dry kibble diet from those that did not, illustrating that healthy feline fecal microbiomes are strongly structured by host diet. ASVs from *Prevotella* and *Megamonas* genera were again identified as features of top importance for predicting if a sample was from a cat who had or had not received a dry kibble diet.

In conclusion, we observed a distinct and significant gut microbiome signature associated with a dry kibble diet in healthy pet cats. Since dry kibble diets are generally higher in carbohydrates than canned diets^14^, it is no surprise that *Prevotella* and *Bifidobacterium* ASVs were found to be enriched, as both genera are known for their ability to break down carbohydrates^15–18^. Both *Prevotella* and *Bifidobacterium* genera include many species that produce enzymes that break down carbohydrates in several mammalian hosts, including cats^17,18^. Furthermore, *Prevotella* and *Megamonas* are important genera in both the ANCOM-BC and random forest analyses. Both *Prevotella* and *Megamonas* spp. were previously found to be enriched in cats infected with an enteric protozoal pathogen that causes chronic colitis and diarrhea^19^, suggesting these genera are generally elevated during feline dysbiosis.

A previous study using fluorescence in situ hybridization quantification appeared to find that *Bifidobacterium* spp. were enriched in healthy cats compared to those with inflammatory bowel disease^20^, potentially contradicting our findings. However, it is important to consider dietary differences in that study, since the cats with inflammatory bowel disease were fed a canned therapeutic gastrointestinal food, while the healthy cats were fed a dry kibble non-therapeutic commercial diet. Thus, higher levels of *Bifidobacterium* in the healthy group likely reflect the influence of a dry kibble diet, aligning with our observation that *Bifidobacterium* spp. are enriched in cats fed dry kibble diets.

The carbohydrate content of cat food has long been debated in veterinary medicine, with no direct known link of high carbohydrate content with any disease. Cats are an understudied species, and we do not currently have enough data in this species to know all of the implications of these results. However, we do have data in several other mammalian species that show consistent trends. *Megamonas* spp. are associated with obesity in both humans and mouse models, where several species appear to promote intestinal lipid absorption^21^. *Megamonas* ASVs were found to be elevated in humans with type 2 diabetes mellitus compared with the control group and treatment with metformin caused a significant reduction in *Megamonas* spp.^22^. That study also found a significant positive correlation between *Megamonas* and blood glucose levels, glycated hemoglobin (A1C), and serum fructosamine levels^22^. Furthermore, there is evidence that healthy cats on high protein, low-carbohydrate diets (more common for wet foods) have improved short-term glucose control with significantly lower fructosamine levels than those on lower protein, high carbohydrate diets (more common for dry foods)^23^. Results from that study also suggested that overweight cats may respond differently to diets than lean cats, which may help explain why diabetic cats, typically obese, respond strongly to low-carbohydrate diets. It is also known that diabetic felines are more likely to achieve remission—no longer requiring insulin or other diabetic medications—on a low carbohydrate, typically canned, diets^24–26^. Weight loss and dietary compliance also significantly contribute to diabetic remission. Thus, enrichment of *Megamonas* ASVs in cats on dry kibble diets may help create a predisposition towards metabolic disease.

While these findings are informative and add to knowledge in the field, there are limitations. One of the biggest limitations in this dataset is the reliance on owner-reported diet histories, medical diagnoses, and health status. Because more specific dietary information is unavailable, we are unable to do correlations with specific micro or macronutrient profiles. However, the variability and partial overlap in signal indicate that macronutrient contents, including different carbohydrate and fiber sources^27^, could be explored to improve our understanding of how diet impacts health via the microbiome. Additional limitations include that we do not know if the commercial diets fed met AAFCO standards, since not enough information was provided. The treat content of the diet was also not evaluated, nor was the fiber, fat, and ash content. Another limitation is that BCS was self-reported by owners and may contain errors. Future work that includes shotgun metagenomics and paired untargeted metabolomics data is recommended to further assess these factors. Additionally, moisture content is a confounding variable potentially contributing to this observation between the two diet formulations and has previously been shown to affect microbial composition^28,29^, so future research controlling for moisture content is also recommended.

## Methods

This is a reanalysis of the existing KittyBiome dataset with the latest bioinformatic methods and a focus on dietary formulation differences. Methods are fully detailed in the original paper^10^, but brief general information is provided here for context.

### Animals

The UC Davis Institutional Animal Care and Use Committee (IACUC) determined that no animal care protocol was required due to using non-invasively collected waste material. This research project was later expanded with the company AnimalBiome, whose customers agreed in writing to participate in research on the cat fecal microbiome and animal health. From August 2015 to May 2021, samples were collected from 1859 cats. In this paper, we examined a subset of 172 samples from client-owned indoor-only cats determined to be healthy using strict criteria. Cats had to be in good physical condition (body condition score of 4–6/9 or BMI ≤ 50), show no signs of illness or have any recent diagnoses, not have received antibiotics in the past year, be between 0.5 and 12 years old, and not be living in a shelter or sanctuary. A single sample of a cat with a BCS of 3 that was present in the original study was excluded from this analysis. Due to preliminary data as part of an initial study, some felines had BMI rather than BCS evaluated. BMI was calculated with the equation: Weight (kg) / Size Index (shoulder height in meters × body length in meters). All information regarding BCS is reported by owners; derived from recent veterinary records. Owners were asked to collect fecal samples in the AM, but opportunistic sampling occurred, and timing was unconfirmed. Clients were asked to fill out a survey on their pet, and responses were recorded. In this analysis, we were only interested in cats on the two primary diet formulations, dry kibble and/or canned wet food. Thus, two subsets of the KittyBiome dataset were used in this analysis: (1) 156 samples that had client answers of yes/no to dry kibble diet; skipped/no answer removed or (2) 77 samples had client answers of yes/no to both dry kibble diet and canned wet food; skipped/no answer for either removed. Please see the original paper for additional details^10^.

### Sample collection

Two mL screw cap tubes that contained 100% molecular grade ethanol and silica beads were sent to study participants for fecal sample collection and returned by mail. Metadata comes from survey responses. Please see the original paper for additional details^10^.

### Sample processing

After DNA extraction using Qiagen DNeasy PowerSoil DNA Isolation Kit (Germantown, MD, USA) and library preparation, the V4 region of 16S rRNA gene was sequenced on a MiniSeq (Illumina, San Diego, CA, USA) using the primer pair 515f to 806r with Golay error-correcting barcodes in accordance with the Earth Microbiome Project protocol. Please see the original paper for additional details^10^.

### Bioinformatic re-analysis

Demultiplexed sequences were provided, quality filtered with deblur (v2021.09), mitochondria and chloroplast reads were removed, and the resulting data were output into a biom-format table. Qiime2 (v2024.5) was used for downstream analysis. Greengenes2 (v2022.10)^11^ was used for taxonomy and phylogeny. Rarefaction depth for alpha diversity metrics was set to 11,800 reads. Shannon (non-phylogenetic) and Faith PD (phylogenetic) were used to estimate alpha diversity. Kruskal–Wallis test with post hoc Dunn’s test was used for significance testing for either the binary kibble yes/no groups or for the three-way grouping (kibble only, canned wet food only, both). Robust center log ratio (rclr) beta diversity metric, RPCA (non-phylogenetic metric)^13^ and phylo-RPCA (phylogenetic metric)^12^ were used for beta diversity analysis using unrarefied read counts (gemelli v.0.0.12). PERMANOVA on the relevant distance matrix is used to determine significance for any Principal Coordinates Analysis (PCoA) shown. Differential abundance method ANCOM-BC^30^ (v3.21) was used to determine the log fold change of relevant genera (lfc > 0.5, *p* < 0.05) associated with diet type. Qurro^31^ (v0.8.0) was used to create a log ratio based on these differentially abundant genera and was grouped by kibble consumption. Significance was determined using Mann–Whitney *U* test with multiple test correction.

### Machine learning approach

To determine whether feline host microbiota data can be differentiated between fed or not fed a dry kibble diet, we employed Random Forest classifiers. These models were trained on a subset of the data (75%) and then evaluated on a held-out validation set (25%) to assess its ability to classify new, unseen samples. We allowed 100 trees to grow for estimation and performed 5 k-fold cross-validations. The State seed value was set to 2024. To assess the significance of the model’s performance, we employed a permutation test. For each cross-validation iteration, the response variable was randomly shuffled, and a new classifier was trained on the corrupted data. The resulting null distribution of Area Under the Receiver Operating Characteristic Curve (AUC) values was used to calculate an empirical *p* value, indicating the likelihood of observing the original model’s performance by chance. The model’s performance was evaluated using the AUC. A higher AUC value indicates better discriminatory power, with 0.5 representing random chance and 1.0 representing perfect classification. The micro-average is where we calculate metrics globally by counting the total true positives, false negatives and false positives. The macro-average is where we calculate metrics for each label and find their unweighted mean. It does not take label imbalance into account. The 95% confidence interval is not reported because we used a binary classification.

## Supplementary information

Supplementary Information

## Data Availability

QIITA study #15475, NCBI: PRJNA1200722 (https://www.ncbi.nlm.nih.gov/bioproject/PRJNA1200722/).

## Code availability

https://github.com/knightlab-analyses/KittyBiome_reanalysis.
