# Supplementary material for: Cats on dry kibble diet have significantly different microbiome than those on canned wet food
Source: npj Vet Sci. 2026 Mar 26;1(1):1. doi: 10.1038/s44433-025-00001-6 (PMC13086222; doi:10.1038/s44433-025-00001-6)
Supplement: Supplementary file 1 — Supplementary Information [file 44433_2025_1_MOESM1_ESM.pdf]

Supplemental Figure

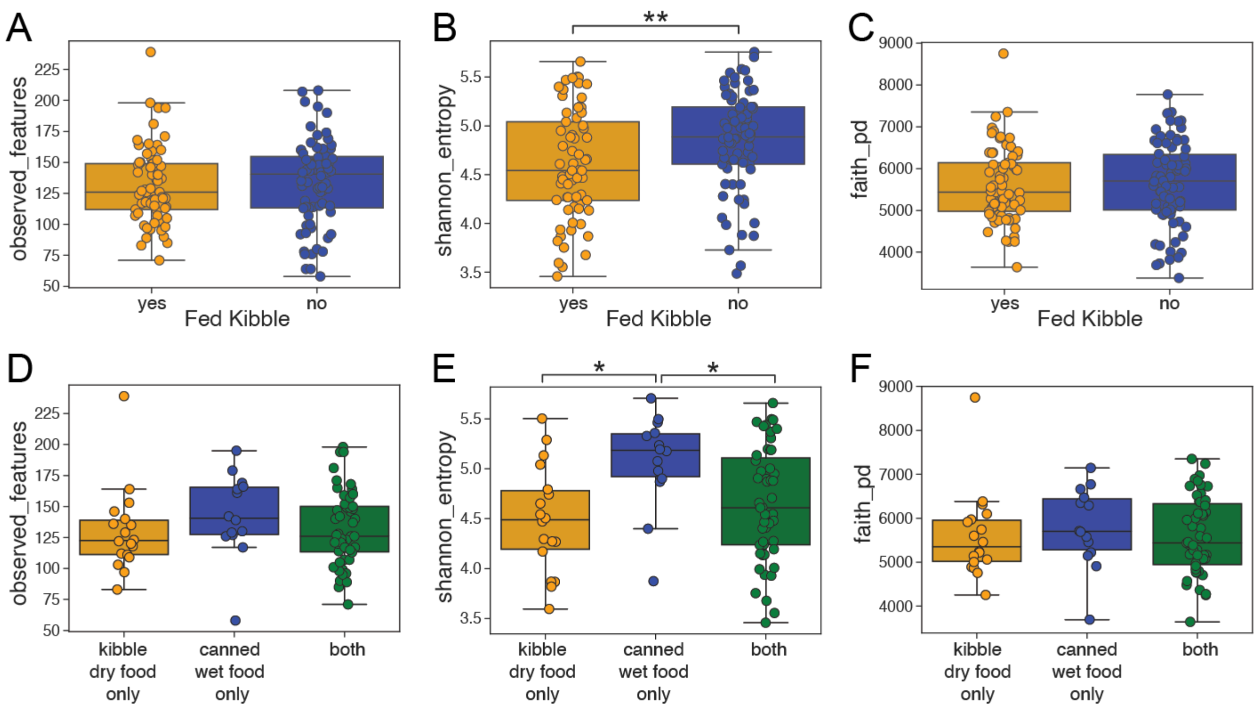

<u>Supplemental Figure 1:</u> Alpha Diversity. For column diet_Dry_kibble, which indicates if client reported if fed any portion of the diet in the day contained a kibble portion or not. A) Non-phylogenetic observed features(ASVs) metric boxplot/stripplot. B) Non-phylogenetic Shannon boxplot/stripplot. C) Phylogenetic Faith's PD boxplot/stripplot. For column diet_form, which indicates if the client reported if fed kibble diet only, canned diet only, or both types of diets. Unknown responses for one or both categories excluded; answer of no/no to both questions also excluded. A) Non-phylogenetic observed features(ASVs) metric boxplot/stripplot. B) Non-phylogenetic Shannon boxplot/stripplot. C) Phylogenetic Faith's PD boxplot/stripplot. Significance determined by Kruskal-Wallis with post hoc Dunn's test. Notation: *= p<0.05, ** = p<0.01.
